# The War and the Rainfall

**Published:** 1915-01-23

**Authors:** 


*To the Editor of* The Hospital.

### The War and the Rainfall.

*To the Editor of* The Hospital.

Sir,—Despite the protestations of meteorological experts many persons are convinced that there is some connection between the recent excessive rainfall and heavy artillery operations across the Channel. The experts put it all down to the working of the law of averages, but it is not unreasonable to attribute some at least of the rain to the war if the following considerations be taken into account.

The firing of explosives results in the formation, among other products, of a considerable amount of aqueous vapour—a ton of cordite is said to give a hundredweight of aqueous vapour; as the combustion of high explosive materials has been proceeding on a hitherto unprecedented scale, the amount of moisture added to the atmosphere in this way must form an appreciable addition to that added in the natural way by evaporation. The mildness of the past autumn may explain the retention of this moisture and its diffusion over a wide area; the cooling of the atmosphere with the onset of winter may be invoked as the cause of its precipitation as rain on either side of the English Channel. If there is anything in this hypothesis the outlook is certainly not cheering for the rest of the winter until the heavens are equal to the strain of retaining extra moisture; nor is it a comforting prospect to contemplate another deluge next December if the war has not terminated. But perhaps the weather experts are right after all.

I venture to inflict these questions and reflections on you, since as a medical man on an enforced holiday, ordered plenty of rest and sunshine, to build me up after an illness, I am forced to ask myself how the prescription ordered for me can be carried out. Since I am precluded from the practical benefits of this prescription by the weather, which has been, as we all know, exceptionally bad, my only relaxation on a "holiday" at a South Coast watering-place is to attempt to diagnose the causes which confine me to my room, with a gale of wind and tempests of rain blowing outside, and making the gentle open-air exercise required by a medical invalid impossible. If any of your readers can throw a scientific light upon the alleged connection of the war and the wet weather I should appreciate it in my exile.—Yours faithfully,

